# Estimating the viral loads of SARS-CoV-2 in the oral cavity when complicated with periapical lesions

**DOI:** 10.1186/s12903-021-01921-5

**Published:** 2021-11-08

**Authors:** Alaa Muayad Altaie, Rania Hamdy, Thenmozhi Venkatachalam, Rifat Hamoudi, Sameh S. M. Soliman

**Affiliations:** 1 Research Institute of Medical and Health Sciences, University of Sharjah, Sharjah, UAE; 2 Department of Physiology and Immunology, College of Medicine, Khalifa University, Abu Dhabi, UAE; 3 Department of Clinical Sciences, College of Medicine, University of Sharjah, Sharjah, UAE; 4 Department of Medicinal Chemistry, College of Pharmacy, University of Sharjah, Sharjah, UAE

**Keywords:** Oral lesions, Fatty acids, SARS-CoV-2, COVID-19, Infection load, Oleic acid

## Abstract

**Background:**

The oral cavity represents a main entrance of the severe acute respiratory syndrome coronavirus-2 (SARS-CoV-2). Angiotensin-converting enzyme 2 (ACE-2), neuropilin-1 (NRP-1), and transmembrane serine protease 2 (TMPRSS2) are essential for the entry of SARS-CoV-2 to the host cells. Both ACE-2 and NRP-1 receptors and TMPRSS2 have been identified in the oral cavity. However, there is limited knowledge about the impact of periapical lesions and their metabolites on the expression of these critical genes. This study aims to measure the impact of periapical lesions and their unique fatty acids (FAs) metabolites on the expression of the aforementioned genes, in addition to interleukin 6 (IL-6) gene and hence SARS-CoV-2 infection loads can be estimated.

**Methods:**

Gene expression of ACE-2, NRP-1, TMPRSS2, and IL-6 was performed in periapical lesions in comparison to healthy oral cavity. Since FAs are important immunomodulators required for the lipid synthesis essential for receptors synthesis and viral replication, comparative FAs profiling was determined in oral lesions and healthy pulp tissues using gas chromatography–mass spectrometry (GC–MS). The effect of major identified and unique FAs was tested on mammalian cells known to express ACE-2, NRP-1, and TMPRSS2 genes.

**Results:**

Gene expression analysis indicated that ACE-2, NRP-1, and TMPRSS2 were significantly upregulated in healthy clinical samples compared to oral lesions, while the reverse was true with IL-6 gene expression. Saturated and monounsaturated FAs were the major identified shared and unique FAs, respectively. Major shared FAs included palmitic, stearic and myristic acids with the highest percentage in the healthy oral cavity, while unique FAs included 17-octadecynoic acid in periapical abscess, petroselinic acid and l-lactic acid in periapical granuloma, and 1-nonadecene in the radicular cyst. Computational prediction showed that the binding affinity of identified FAs to ACE-2, TMPRSS2 and S protein were insignificant. Further, FA-treated mammalian cells showed significant overexpression of ACE-2, NRP-1 and TMPRSS2 genes except with l-lactic acid and oleic acid caused downregulation of NRP-1 gene, while 17-octadecynoic acid caused insignificant effect.

**Conclusion:**

Collectively, a healthy oral cavity is more susceptible to viral infection when compared to that complicated with periapical lesions. FAs play important role in viral infection and their balance can affect the viral loads. Shifting the balance towards higher levels of palmitic, stearic and 1-nonadecene caused significant upregulation of the aforementioned genes and hence higher viral loads. On the other hand, there is a reverse correlation between inflammation and expression of SARS-CoV-2 receptors. Therefore, a mouth preparation that can reduce the levels of palmitic, stearic and 1-nonadecene, while maintaining an immunomodulatory effect can be employed as a future protection strategy against viral infection.

## Background

Coronavirus disease 2019 (COVID-19) outbreak caused by severe acute respiratory syndrome coronavirus-2 (SARS-CoV-2) is a global threat [1]. Oral cavity is considered as an important entry port for the virus [2]. Since COVID-19 seems to stay for longer time, the relationship between oral health and SARS-CoV-2 infection could provide important information to help on making a managing decision related to this contagious disease. On the other hand, the complications of SARS-CoV-2 infection by pathological conditions at the oral cavity such as periapical lesions have never been reported. Viral infection loads via the oral cavity are significantly controlled by the expression of cell membrane binding receptors and host proteases. Studies highlighted the expression of angiotensin-converting enzyme 2 (ACE-2) in the oral mucosa, tongue [3, 4], buccal, gingival tissues [4], in addition to the normal and inflamed dental pulp [5]. Besides, several studies have demonstrated the role of neuropilin-1 (NRP-1) as a receptor for SARS-CoV-2 infection [6–8]. NRP-1 receptor localized to the outer supra-basal epithelial layers of the normal tongue [9] and plays indispensable roles in wound healing, angiogenesis, bone formation, teeth formation [10–13], and odontoblastic differentiation of dental pulp stem cells [14]. Human host proteases particularly transmembrane serine protease 2 (TMPRSS2) enzyme is essential for the activation of SARS-CoV-2 S protein in human airway epithelial cells [15]. TMPRSS2 is highly expressed in oral stratified squamous epithelium, saliva, and tongue tissues [16].

Metabolites of the human body particularly FAs also play an important role in viral infection [17, 18]. Some metabolites present in the human body play a protective role against viral infection including SARS-CoV-2 [19], while others worsen the condition [20]. Metabolites profile in pathological conditions is different than in healthy conditions. These metabolites can develop a microenvironment that may affect the viral infection. Here in this study, the expression of ACE-2, NRP-1, and TMPRSS2 genes in association with FAs microenvironment were investigated in periapical lesions compared to healthy pulp tissues. The effects of both the expression of the aforementioned genes and the FAs microenvironment on SARS-CoV-2 infection load in the oral cavity were then estimated.

## Methods

### Cell culture and metabolites

Human embryonic kidney (HEK293) cells were purchased from (ATCC® CRL1573™, USA) and cultured in Dulbecco's Modified Eagle Medium/Nutrient Mixture F-12 (DMEM/F-12) (Sigma-D8437, Germany), supplemented with FBS (Sigma, Germany), and penicillin–streptomycin (Sigma, Germany). HEK293 cells were employed as model cells that normally express both ACE-2 and NRP-1 receptors and TMPRSS2 enzyme and related to high comorbidity in SARS-CoV-2 infection. The purchased metabolites were palmitic acid (Sigma Aldrich, Germany), stearic acid (Sigma Aldrich, Germany), oleic acid (Daejung, Korea), 17-octadecynoic acid (Santa Cruz, USA), 1-nonadecene (TCI, US-Japan), and l-lactic acid (Sigma Aldrich, Germany).

### Sample’s collection

This study includes 40 samples of 28 males and 12 females with an age range of 15–60-year-old. The samples were 10 periapical abscesses, 10 preapical granulomas, 10 radicular cysts, and 10 healthy pulp tissues, employed as healthy control. For all periapical lesions, the general inclusion criteria included carious lesion, necrotic pulps, evidence of periradicular radiolucency and bone loss, in addition to facial pain and swelling in cases of periapical abscess [21]. The exclusion criteria included systemic diseases, history of corticosteroid treatment, pregnancy, radiotherapy, tooth mobility, and vertical tooth fracture. The sampling procedure was performed as previously described [22, 23]. Periapical lesions were isolated from the root tips using sterile surgical scalpel blade No.11. Healthy pulp tissue samples were collected after surgical extraction of impacted wisdom teeth and as previously described [24, 25].

The collected samples were confirmed by standard histopathology procedure following fixation in 10% neutral buffered formalin for at least 24 h and subsequent embedding in paraffin wax. The histopathological structure of the lesions was assessed by traditional haematoxylin and eosin staining for optical microscope examination. Periapical granulomas and radicular cysts were differentiated according to the absence or the presence of a clear cavity lined by epithelium [26].

### Fatty acids (FAs) profiling in oral lesions

Compared to healthy control, FA metabolites profiling in oral lesions were identified following a previously described method [27, 28]. Briefly, frozen tissues were suspended in methanol: chloroform (1:1) followed by sonication in a sonicator water bath for 1 h at room temperature. The filtered extracts were derivatized and analyzed by GC–MS following protocol established by Soliman et al., 2020 [27]. The metabolite extracts were derivatized by mixture of *N*-Trimethylsilyl-*N*-methyl trifluoroacetamide and Trimethylchlorosilane (MSTFA + 1% TMS) and analyzed by GC–MS using QP2010 gas chromatography–mass spectrometer (GC-2010 coupled with a GC–MS QP-2010 Ultra) equipped with an auto-sampler (AOC-20i + s) from Shimadzu (Tokyo, Japan), using Rtx-5 ms column (30 m length × 0.25 mm inner diameter × 0.25 µm film thickness: Restek, Bellefonte, PA). FAs data were extracted from the metabolites list identified using NIST library. The average % amount of each metabolite was identified in relation to other metabolites per sample. Fold difference of each metabolite in relation to healthy control was displayed. A comprehensive search was performed to correlate the identified FA to viral infection particularly those related to coronavirus including COVID-19 infection (Table 1).

**Table 1:** Metabolite microenvironment of oral lesions in relation to viral infection particularly coronavirus including SARS-CoV-2

Metabolite	Class	Detected levels of metabolites in oral cavity				Reported levels of metabolites during coronavirus infection	Reported effect of metabolites on coronavirus infection	Reported antiviral activity of metabolites	Reported effect of metabolites on IL-6
		Oral lesions			Healthy control				
		Periapical abscess	Periapical granuloma	Radicular cyst					
Palmitic acid	Saturated	6.06	3.28	9.51	13.51	Increased [29]	Decrease the infection [30]	Weak antiviral [31]	Increase [32]
Stearic acid		5.99	4.40	7.54	11.70	Increased [29]	NR	Weak antiviral [31]	Increase [33, 34]
Myristic acid		0.24	0.19	0.18	0.26	Increase [35, 36]	NR	Antiviral [37]	Decrease [38]
Octanoic acid		0.00	0.09	0.00	0.00	NR	NR	Weak antiviral [39]	No effect [40]
l-lactic acid		0.00	1.48	0.00	0.00	Increased [41]	Ongoing clinical trial [42]	Antiviral [43]	Decrease [44, 45]
Oleic acid (monounsaturated omega-9 fatty acid)	Monounsaturated	0.00	0.00	0.00	1.00	Increased [29, 46]	Enhances phagocytic activity of macrophages [47] Inhibit ACE-2 receptors [48]	Antiviral [31, 49]	Decrease [50]
17-Octadecynoic acid		0.35	0.00	0.00	0.00	NR	NR	NR	No effect [51]
Petroselinic acid		0.00	0.16	0.00	0.00	NR	NR	Has binding capacity to RBD of coronavirus and to ACE-2 [52]	Anti-inflammatory [53]
1-Nonadecene		0.00	0.00	0.24	0.00	NR	NR	NR	NR

*NR* not reported

### Calculation of FA protein binding affinity

The 3D structures of the selected FAs were downloaded as SDF file from PubChem database, followed by energy minimization and saved as PDBQT format. The 3D crystal structures of proteins were downloaded from Protein Data Bank (PDB) (http://www.rcsb.org). The proteins were prepared, and hydrogen was added, while water, ligand and hetero ions were removed. The proteins were converted to macromolecules PDBQT format with Chimera software before docking. The binding affinity of FAs with the target proteins was calculated using Pyrx Autodock binding engines. The acids metabolites were docked within the binding site of the ACE-2 (PDB:1R4L), TMPRSS2 (PDB: 2OQ5) and S protein (PDB: 6MOJ) using Autodock Vina as docking engines.

### Treatment of mammalian cells with FAs

HEK293 cells were cultured in DMEM/F-12 supplemented with 10% FBS, 1% penicillin–streptomycin and maintained at 37 °C and 5% CO_2_. FA metabolites were dissolved in DMSO to prepare a stock of 1 mg/ml and then diluted with PBS to get the desired concentrations. The cells were treated with 10 ng/ml and 1000 ng/ml of palmitic acid, stearic acid, oleic acid, 17-octadecynoic acid, 1-nonadecene, and l-lactic acid for 48 h. The cells were then harvested and kept at − 20 °C for downstream experiment.

### Quantitative real-time PCR (qRT-PCR)

Clinical samples and treated HEK293 cells were used to quantify the gene expression of ACE-2, NRP-1 TMPRSS2, and IL-6transcripts. RNAs from homogenised samples and treated cells were isolated using RNeasy Mini Kit (QIAGEN, Germany), then reverse transcribed to cDNA using SuperScript™ III first-strand synthesis system (ThermoFisher Scientific, USA) according to manufacture instructions. Primer sequences are listed in Table 2. RT-PCR setup and cycling procedures were conducted using QuantStudio3 as previously described [54]. GAPDH was used as a housekeeping gene for normalization and the relative fold change was calculated using 2^^−(ΔΔCt)^.

**Table 2:** Primer sequences employed in this study

Gene	Forward (5′–3′)	Reverse (5′–3′)	Reference
ACE2	TCCGTCTGAATGACAACAGC	CACTCCCATCACAACTCCAA	[55]
NRP-1	CCCAACAGCCTTGAATGCAC	ATTTCTAGCCGGTCGTAGCG	[56]
TMPRSS2	GTCCCCACTGTCTACGAGGT	CAGACGACGGGGTTGGAAG	[57]
IL-6	AGACAGCCACTCACCTCTTCAG	TTCTGCCAGTGCCTCTTTGCTG	[58]
GAPDH	GTCTCCTCTGACTTCAACAGCG	ACCACCCTGTTGCTGTAGCCAA	[59]

### Statistical analysis

The collected data were organized in Excel worksheet and analysed using GraphPad (version 5.01). One-way or two-way analysis of variance (ANOVA) were used to analyse the quantity of the extracted metabolites and gene expression as indicated per each graph. The fold change of metabolites was calculated to compare the level of metabolites (area%) between lesions and healthy control, or within a group. Oleic acid in healthy control was represented as a reference for calculating the approximate fold value for the other metabolites in all groups (Table 1).

## Results

### Expression of ACE-2, NRP-1 and TMPRSS2 genes were higher in healthy rather than periapical lesioned oral cavity, while IL-6 gene expression was the reverse

**Fig. 1:**
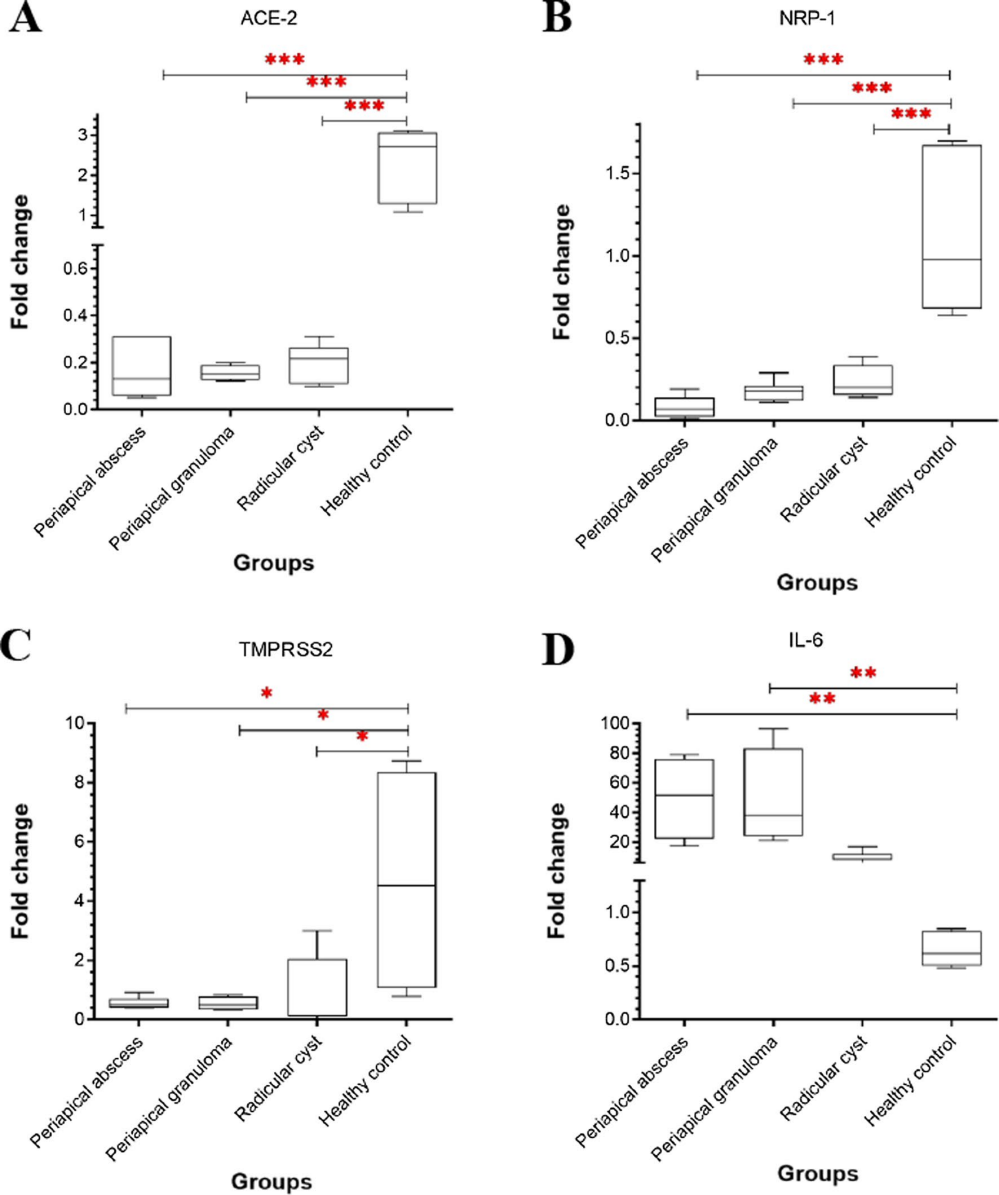
Quantitative gene expression in periapical lesions and healthy controls. Identified gene expression was displayed as fold changes between groups. **A** ACE2. **B** NRP-1. **C** TMPRSS2. **D** IL-6. The data was analyzed using one-way analysis of variance (ANOVA), the statistical significance was calculated using Bonferroni’s multiple comparisons test and the significance level indicated by asterisks (*P < 0.05; **P < 0.01: ***P < 0.001; ****P < 0.0001). *P-value* < 0.05 was considered as significant. The data display the fold change in gene expression of10 samples

To expect an estimated load of viral infection in oral lesions compared to healthy control, the expression of ACE-2, NRP-1, and TMPRSS2 genes in clinical samples was analysed. The results obtained indicated that ACE-2 and NRP-1 gene expression were significantly downregulated in periapical abscess, periapical granuloma, and radicular cyst when compared to healthy control (*P* ˂ 0.001) (Fig. 1A, B). Additionally, the same pattern was observed for the expression of TMPRSS2 (*P* ˂ 0.05) (Fig. 1C). These results indicate that the expression of critical genes required for viral infection was significantly reduced in oral lesions compared to healthy control. However, the expression of IL-6 gene was upregulated significantly (*P* ˂ 0.01) in periapical abscess and granuloma but insignificantly in radicular cyst compared to healthy control (Fig. 1D).

### FAs of oral lesions were lower than those of healthy control

Fatty acids (FAs) are one of the major metabolite classes that affect the microenvironment of the oral cavity and hence the infection rate [60, 61]. FAs also play an important role in lipid synthesis required for viral replication [17, 18]. Therefore, a comparative FAs analysis of oral lesions versus healthy oral cavity was conducted. The microenvironment of the identified FAs in oral lesions was compared to healthy control, and then correlated to the expression of receptors, TMPRSS2 activator enzyme, and IL-6 immunomodulator and hence to the SARS-CoV-2 infection loads in the oral cavity.

Qualitative and quantitative metabolomics analyses indicated that FAs were highly present in oral healthy control than in periapical oral lesions (Table 1). Palmitic acid, stearic acid, and myristic acid represented the most abundant saturated FAs identified in healthy control than in lesions. Literature’s search showed that the aforementioned FAs are increased during coronavirus infection [29, 35, 36]. Reports also indicated that both palmitic and stearic acids have week antiviral activity [31]. Oleic acid, a monounsaturated FA, has been also reported at a high level during coronavirus infection, while it is known to be accompanied by significant antiviral [31, 49] and anti-inflammatory effects [50]. Similarly, l-lactic acid, which is exclusively present in periapical granulomas, was also reported at a higher amount during coronavirus infection [41] and is known to exert antiviral [43] and anti-inflammatory effects [44, 45].

### Docking study indicated that the identified FAs were less likely bind to target proteins

To test the ability of the identified FAs to inhibit critical proteins in virus infection, molecular docking of FAs to ACE-2, TMPRSS2, and S protein was performed. The results showed a lower binding affinity of the identified FAs when compared to standards ligands including enalaprilat, benzamidine, and camostat (Table 3).

**Table 3:** Binding affinity of identified metabolites to ACE2, TMPSS2, and S protein

Metabolites	Binding affinity (Kcal/mol)		
	ACE2	TMPRSS2	S protein
Palmitic acid	− 5.7	− 4.5	− 5.5
Stearic acid	− 5.6	− 4.9	− 5.1
Myristic acid	− 5.6	− 4.2	− 5.2
Oleic acid	− 5.6	− 5.0	− 4.9
17-Octadecynoic acid	− 5.6	− 5.3	− 5.6
Petroselinic acid	− 6.0	− 5.1	− 5.4
Octanoic acid	− 4.8	− 4.5	− 4.6
1-Nonadecene	− 4.9	− 4.3	− 4.8
l-(+)-lactic acid	− 4.0	− 4.1	− 4.1
Enalaprilat	− 8.8	–	–
Benzamidine	–	− 5.6	–
Camostat	–	− 7.7	–
Linoleic acid	–	–	− 5.6

### FAs of healthy oral cavity enhanced the expression of ACE-2, NRP-1 and TMPRSS2 genes

**Fig. 2:**
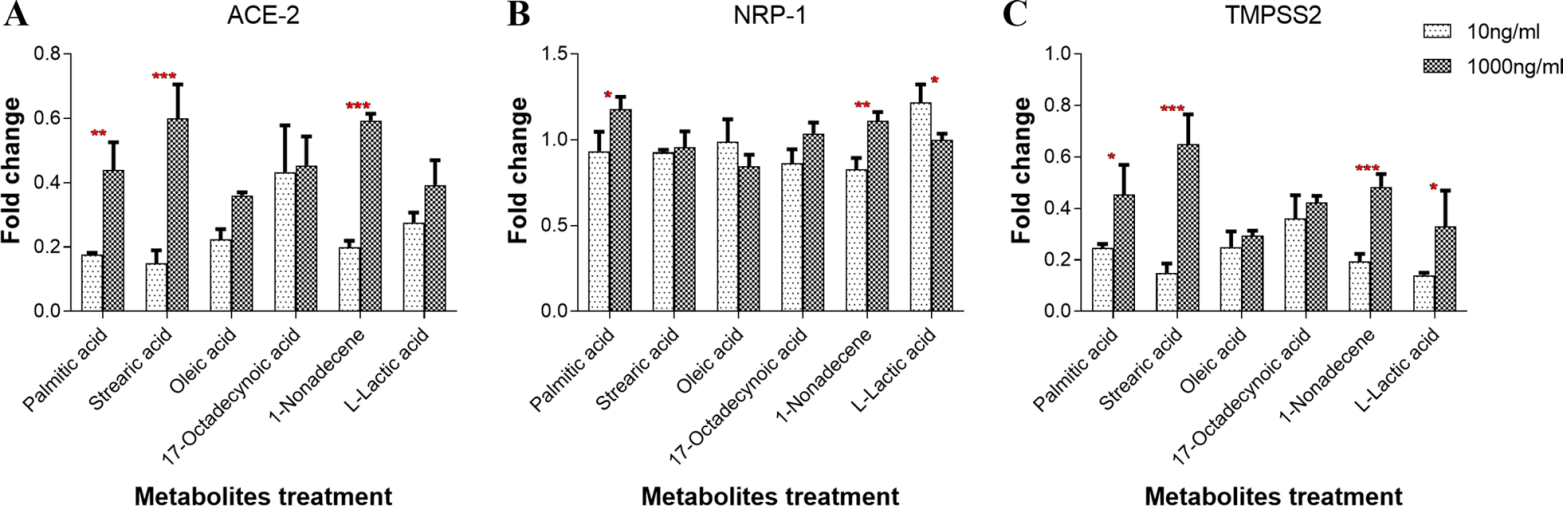
Quantitative gene expression of treated HEK293 cells. HEK293 cells were treated with two different concentrations of 10 ng/ml and 1000 ng/ml of palmitic acid, stearic acid, oleic acid, 17-octadecynoic acid, 1-nonadecene, and l-lactic acid for 48 h. **A** ACE2. **B** NRP-1. **C** TMPSS2. Gene expression of the treated cells was normalised with its corresponding vehicle values. The data were analysed using two-way ANOVA, the statistical significance was calculated using Bonferroni’s multiple comparisons test and the significance level indicated by asterisks (*P < 0.05; **P < 0.01; ***P < 0.001; ****P < 0.0001). *P-value* < 0.05 was considered as significant. The data display the fold change in gene expression in 3 replicas

To validate the importance of FAs in viral infection loads, HEK293 cells, employed as model cells endogenously expressing ACE-2, NRP-1, and TMPRSS2 were treated with selected FAs that were significantly differ between lesions and healthy control (Table 1). Two different concentrations of the tested metabolites, representing the difference between lesions and healthy control, were used. The results showed a significant increase in the gene expression of ACE-2 transcript in relation to the increase in concentration of palmitic acid (*P* ˂ 0.01), stearic acid (*P* ˂ 0.001), and 1-nonadecene (*P* ˂ 0.001) (Fig. 2A). Other FA metabolites including oleic acid, 17-octadecynoic acid, and l-lactic acid insignificantly upregulate the expression of ACE-2 at the higher concentrations (Fig. 2A). Furthermore, the expression of NRP-1 gene was upregulated at higher concentrations of palmitic acid (*P* ˂ 0.05) and 1-nonadecene (*P* ˂ 0.01), and at a lower concentration of l-lactic acid (*P* ˂ 0.05) (Fig. 2B). Insignificant expression of NRP-1 was observed when the cells were treated with stearic acid, oleic acid, and 17-octadecynoic acid (Fig. 2B). TMPRSS2 gene expression was also conducted and showed upregulation at higher concentrations of all tested FAs especially and significantly with palmitic acid (*P* ˂ 0.05), stearic acid (*P* ˂ 0.001), 1-nonadecene (*P* ˂ 0.001), and l-lactic acid (*P* ˂ 0.05) (Fig. 2C). On the other hand, the expression of ACE-2, NRP-1 and TMPRSS2 was insignificantly affected when the cells were treated with higher concentrations of oleic acid or 17-octadecynoic in comparison to other FAs. This is in accordance with the data reported in Table 1.

**Fig. 3:**
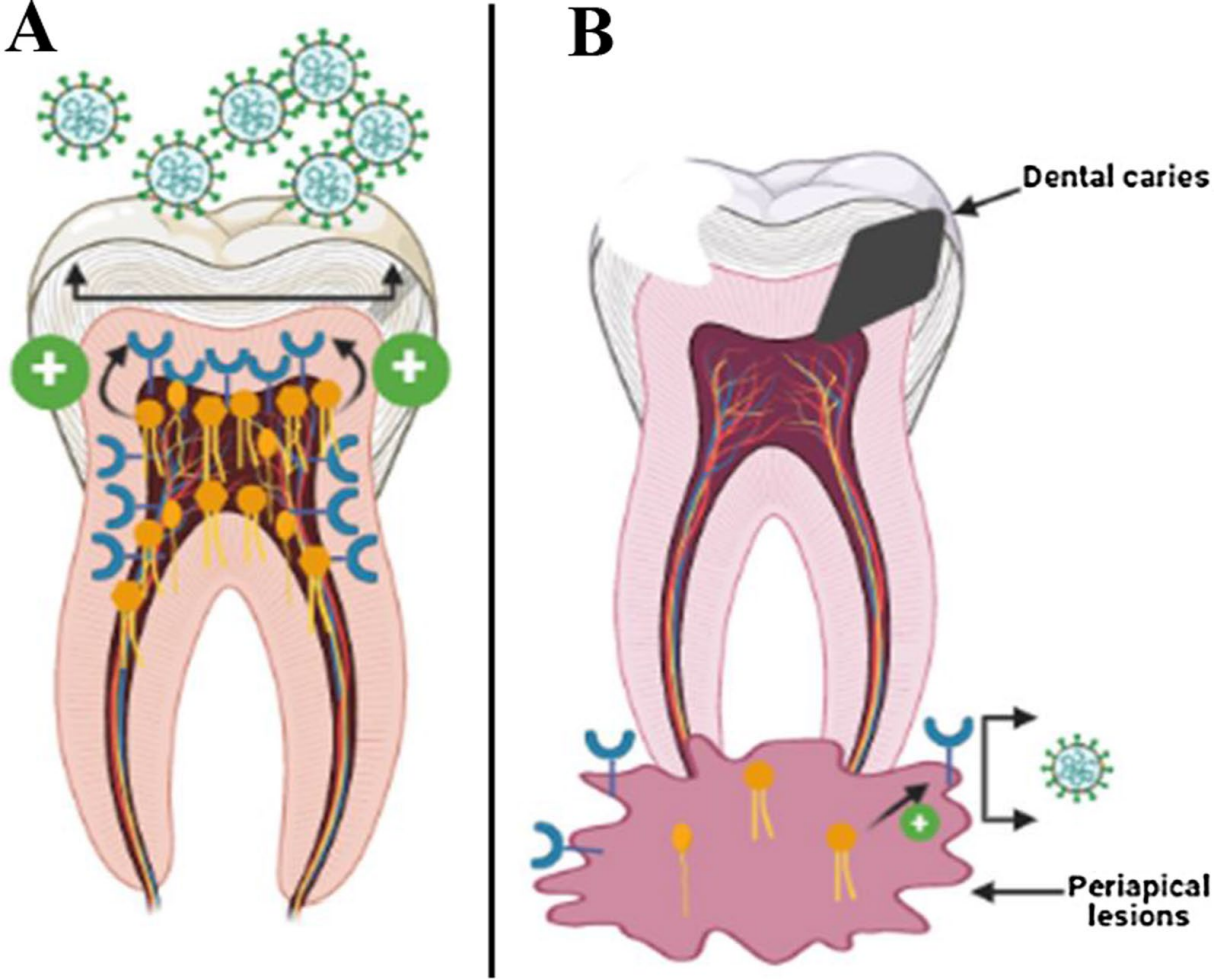
Summarized estimation of viral infection loads in healthy pulp tissues versus periapical lesions. **A** In healthy pulp tissues, FAs (indicated in orange color) are existed in higher amounts, which in turn increased the expression of host receptors (indicated in blue color), leading to increased viral infection loads. **B** In periapical lesions, low level of FAs accompanied with lower expression of host receptors and hence reduces the viral infection load

Collectively, higher concentrations of FAs particularly palmitic acid caused significant upregulation of the expression of ACE-2, NRP-1, and TMPRSS2 genes, while stearic acid significantly upregulated ACE-2 and TMPRSS2 gene expression. This is suggesting a higher viral load in the case of healthy compared to lesioned oral cavity (Fig. 3). The data were in accordance with those represented in Table 1 and Fig. 1A–C. Furthermore, 1-nonadecene significantly upregulated the gene expression of ACE-2, NRP-1, and TMPRSS2, indicating a possible higher viral load in radicular cysts compared to other periapical lesions but less than the healthy control. In reverse, inflammatory responses measured by IL-6 gene expression was higher in periapical abscess and granuloma than radicular cysts and healthy control (Fig. 1D). On the other hand, oleic acid and l-lactic acid reduced the expression of NRP-1 gene.

## Discussion

**Fig. 4:**
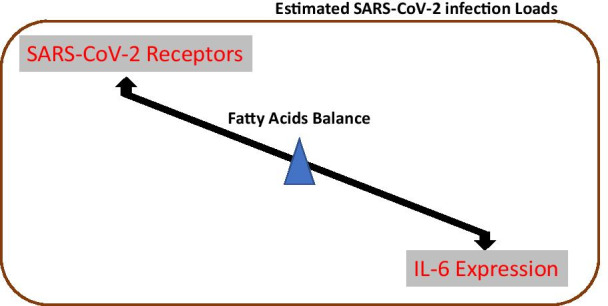
Model representing the effect of reverse correlation between the expression of SARS-CoV-2 receptors and IL-6 on viral infection loads in periapical lesions compared to healthy oral cavity

SARS-CoV-2 infection can be complicated by pathological conditions including those related to the entry of the virus at the oral cavity such as periapical lesions. Oral cavity expresses critical protein receptors such as ACE-2 and NRP-1 and enzymes such as TMPRSS2 required for viral entry and infection. Therefore, the impact of periapical lesions on the viral infection loads was studied in comparison to healthy oral cavity. Our results indicated that the expression of ACE-2, NRP-1 and TMPRSS2 was significantly reduced in oral lesions compared to healthy control. To investigate a reason for that, FAs microenvironment of the oral lesions compared to healthy control was studied since FAs play an important role in lipid rafts synthesis and hence receptors expression [18], in addition to their role in viral assembly [62] and host immunomodulatory effect. The results showed that FAs including palmitic acid, stearic acid, and myristic acid were highly presented in the oral healthy control than in periapical oral lesions. Furthermore, palmitic acid, and stearic acid-treated mammalian cells exhibited higher expression of ACE-2, NRP-1 and TMPRSS2, while oleic acid and l-lactic acid reduced the expression of NRP-1 and insignificantly affect the expression of ACE-2. This is in accordance with previously reported data on other coronaviruses as summarized in Table 1. On the other hand, there is a reverse correlation between the expression of IL-6 and SARS-CoV-2 receptors (Model represented in Fig. 4). These data estimated a higher viral infection load in healthy compared to periapical lesioned oral cavity. Therefore, an adverse and high cross-infectivity for the dental health workers may be expected during the dental procedures on healthy teeth in patients with COVID-19 infection. The results obtained can propose a special precautionary measure and safe environment to prevent the spread of disease during oral cavity-related procedures to be included in a general dental guideline [63, 64].

The expression of ACE-2, and NRP-1 in our study was higher in healthy pulp tissue than in periapical oral lesions, which is different than the results reported in the case of oral cancer, another oral pathological condition. ACE-2 [65] and NRP-1 [9] gene expression was reported at higher levels in oral cancer tissues. The difference with our results may be attributed to a variation in other factors including in particular inflammatory responses [66]. In accordance, our results in Fig. 1 showed a reverse correlation between IL-6 and receptors expression, which is consistent with previously reported data on ACE-2 [67, 68] and NRP-1 [69] expression in relation to inflammatory responses. Besides, oleic acid and l-lactic acid are known immunomodulators [70] that can downregulate receptor expression. Similar to our results, the expression of TMPRSS2 gene is lower in the oral cavity of the head and neck squamous cell carcinoma [71], confirming that other factors such as inflammatory responses may be involved.

In this study, FAs levels were assessed in normal versus pathological oral cavity to predict the load of SARS-CoV-2 infection. FAs metabolism has been reported to be highly correlated to COVID-19 cases providing insights about their roles in either potentiating or reducing the infection [72]. Our results showed that saturated FAs such as palmitic and stearic acids were highly present in the healthy pulp tissue and were able to upregulate the expression of SARS-CoV-2 receptors in in vivo (Fig. 1) and in vitro (Fig. 2). These results may indicate their roles during the viral infection since they have been previously reported in high levels during coronavirus infection [29]. Both FAs are known to increase sphingolipids synthesis and the formation of lipid rafts and hence increase the expression of ACE-2 receptors [18]. Besides, both FAs possess weak antiviral activity [31] and proinflammatory effects [32–34].

In contrast, l-lactic acid was the exclusive metabolite detected in the periapical granuloma. Treatment of mammalian cells with l-lactic acid caused insignificant upregulation of ACE-2 (Fig. 2A) gene expression but significant downregulation of NRP-1 (Fig. 2B). Compared to healthy control, the expression of SARS-CoV-2 receptors and IL-6 in periapical granuloma remained significantly lower and higher, respectively (Fig. 1). This is indicating a downregulation of receptor expression due to inflammation, leading to an unfavourable binding condition for the virus in the periapical granuloma. In consistence, l-lactic acid has been reported at high level during coronavirus infection [41]. On the other side, l-lactic acid possesses antiviral [43] and anti-inflammatory effects [44, 45].

The effect of unsaturated FAs was different. 17-Octadecynoic acid was the only monounsaturated FA reported in periapical abscesses. The impact of 17-octadecynoic acid on coronavirus infection has never been reported. Mammalian cells treated with 17-octadecynoic acid caused insignificant effect on the expression of SARS-CoV-2 receptors. However, periapical abscesses showed a significant low expression level of SARS-CoV-2 receptors and higher expression of IL-6. These results indicated that periapical abscess is exposed to a lower viral infection load. On the other hand, 1-nonadecene has been identified for the first time as the exclusive unique unsaturated metabolite in the radicular cyst. Although there is a gap of knowledge regarding the biological activity of this metabolite, our study showed significant upregulation of SARS-CoV-2 receptors, which is inconsistent with the in vivo study (Fig. 1A–C). The difference in the expression of SARS-CoV-2 receptors between the in vitro (metabolite-treated mammalian cells) and in vivo (oral lesions) studies may be attributed to the effect of other factors such as the inflammatory responses (Fig. 1D). Furthermore, unsaturated FAs such as petroselinic acid [52], and oleic acid [31, 49] are known to have antiviral activity.

Computationally, the binding affinity of the identified FAs to critical proteins and enzymes required for SARS-CoV-2 infection was low; hence, experimental testing still needs to be performed compared to linoleic acid. Linoleic acid has been reported as an excellent blocker of S protein by exerting conformational changes on S protein [73], although it showed a similar binding affinity to the FAs identified in this study.

In conclusion, oral lesions including periapical abscess, periapical granuloma, and radicular cyst showed lower expression of critical genes required for SARS-CoV-2 infection, predicting a lower viral infection load compared to healthy pulp tissues. The results obtained also indicated the superior effect of saturated FAs such as palmitic and stearic acids on the expression of the aforementioned genes. The balance between FAs in the oral cavity may be a detrimental factor for the expression of protein receptors and enzymes required for the viral infection. Shifting the balance towards saturated FAs such as palmitic and stearic acids increased the expression of viral receptors and hence the viral load as in the case of healthy pulp tissues. Furthermore, immunomodulators such as oleic and l-lactic acids can reduce the expression of SARS-CoV-2 receptors and hence the viral loads. For instance, a mouth preparation that can reduce the level of palmitic and stearic acids and with immunomodulatory effect can be employed as a preventive agent against viral infection.

## Data Availability

All data generated or analysed during this study are included in this published article.
